# Acromioclavicular Reconstruction using Autogenous Semitendinosus Tendon Graft and the Importance of Postoperative Rehabilitation: A Case Report

**DOI:** 10.5704/MOJ.1311.012

**Published:** 2013-11

**Authors:** Jade PY Ho, A Ahmad Faizal, N Sivapathasundaram

**Affiliations:** Department of Orthopaedics, Malacca General Hospital, Malacca, Malaysia; Department of Orthopaedics, Malacca General Hospital, Malacca, Malaysia; Department of Orthopaedics, Malacca General Hospital, Malacca, Malaysia

## Abstract

We present a case of chronic acromioclavicular joint dislocation (Rockwood type 5) in which the choice of acromioclavicular reconstruction using autogenous semitendinosus tendon graft was made due to its superiority in anatomical reconstruction of the coracoclavicular ligaments, and the impact of postoperative rehabilitation on the recovery of this patient. We also discuss the rationale behind this.

**Key Words:**

acromioclavicular joint dislocation, acromioclavicular reconstruction, autogenous semitendinosus tendon graft, rehabilitation approach

## Introduction

Acromioclavicular joint (ACJ) dislocations have been approached differently in terms of surgical management. In acute cases, reduction and stabilization of the ACJ, usually via hardware fixation, allows for primary healing as the ligaments are held in anatomical apposition. However, if it is a chronic injury, a reconstructive procedure is often required, one of the most commonly done being the modified Weaver- Dunn procedure. There is no gold standard in ACJ reconstruction (ACJR) and many methods have been described in literature, with variable outcomes. In recent years, a technique using autogenous semitendinosus tendon graft has been advocated and proven to be superior biomechanically, clinically and radiologically compared to more conventional methods^1^,^2^. It is also important to note that postoperative rehabilitation after reconstruction can determine the outcome of surgery.

## Case Report

A 29-year old male presented to our outpatient department three months after being involved in a motor vehicle accident in which he fell off his motorcycle and landed on his left shoulder. He complained of persistent left shoulder pain which did not go away despite rest and analgesics. On examination, the distal part of the left clavicle was noted to be prominent and there was mild tenderness over the acromioclavicular joint (ACJ). He was unable to abduct his left shoulder beyond 90 degrees. Radiographic studies of the left shoulder revealed a disrupted ACJ (Rockwood type V; see Figure 1(a)). The patient agreed for surgery two months later but unfortunately it was delayed until one year post trauma due to unavoidable circumstances.

Anatomical reconstruction of the coracoclavicular ligament was performed using autogenous semitendinosus tendon graft with bioscrew fixation. General anaesthesia was administered and the patient was placed in a beach-chair position. Semitendinosus tendon graft was harvested from the left knee. A strap incision was used to approach the area of interest. The muscle/fascia plane of the deltoid and trapezius was then developed to expose the clavicle. The coracoid process was also identified. Subsequently, bone tunnels were created at the base of the coracoid process and clavicle; one 30 mm from the ACJ in the centre of the clavicle, the other 45 mm from the ACJ, more posteriorly. The tendon graft was then passed from the tunnel in the coracoid process and secured with a 6 mm x 23 mm bioscrew. Each limb was passed to the respective tunnels in the clavicle and the medial limb was sutured to the lateral limb. Finally, the lateral limb was used to bridge the ACJ and secured to it. The wound was irrigated and closed in layers.

Immediate postoperative radiograph showed a reduced ACJ with a non-displaced distal clavicle fracture which was not apparent on preoperative radiographs (Figure 1(b)). The patient was instructed to use an arm sling for six weeks and to apply ice to reduce swelling. Pendulum exercises were started thereafter by the sports physician and range of motion exercises started at eight weeks post-op. At twelve weeks post-surgery, strengthening exercises were taught to the patient. Pain over the left shoulder was minimal and there were no reported complaints with regards to the donor site (left knee). Serial radiographs were taken to check on the reduction of the ACJ.

The patient appeared to make good progress but unfortunately, by the tenth week post-op, the shoulder AP radiograph showed subluxation of the ACJ (Figure 2(a)). Nevertheless, he did not complain of pain and we advised the patient to continue performing the exercises we had taught him as mentioned above. Radiograph taken at five months post-operatively showed a dislocated ACJ with a displaced fracture (Figure 2(b)). At this point, he was able to achieve abduction up to 140 degrees, forward flexion up to 150 degrees, internal rotation up to the tip of scapula and full range of motion for external rotation (Figure 3). There was some tenderness on palpation of the ACJ.

When questioned further, the patient had also been concurrently visiting the physiotherapist, whom he was referred to upon his initial presentation to us. The physiotherapist had prescribed strengthening exercises to our patient at two weeks post-surgery. He was compliant and continued to perform these exercises throughout, which very likely caused the construct failure. This patient is still under our follow-up and is planned for revision surgery soon.

## Discussion

ACJ reconstruction (ACJR) using tendon grafts is not a new technique, but is less popular than more conventional methods such as the modified Weaver-Dunn procedure possibly due to it being more technically demanding, requiring an extra procedure for tendon harvest, or if cadaveric grafts are used, the high cost associated with it. Numerous complications have been reported with the modified Weaver-Dunn procedure and it has also been criticized for placing the clavicle in a non-anatomical position. Therefore, anatomical ACJR was designed to place tendon grafts at the exact anatomic location of the coracoclavicular (CC) ligaments and to reconstruct the ACJ capsule.

Besides avoiding the need for implant removal and complications such as hardware migration, infection, and foreign body reaction, ACJR using tendon grafts is biomechanically superior to coracoacromial ligament transfers. This was demonstrated by Lee et al^1^ in a controlled laboratory study, whereby reconstruction using coracoacromial ligament transfer with and without augmentation, and a semitendinosus tendon were subjected to two loading cycles. Only the semitendinosus tendon survived both. Clinically this should translate to a strong and stable biologic option for ACJR.

Tauber et al2 set out to confirm the clinical relevance of the more superior biomechanical properties of the semitendinosus tendon graft and they found that semitendinosus tendon graft for CC ligament reconstruction (CCR) resulted in significantly superior clinical and radiologic outcomes compared to the modified Weaver-Dunn procedure. In addition, CCR with an autogenous hamstring tendon graft was also shown to be satisfactory in treating painful non-united distal clavicle fractures with CC separation^3^. Therefore, in our case, the missed distal clavicle fracture (which could also be a complication from the surgery) would have been treated by this method as well.

Postoperative rehabilitation is another important aspect in determining the outcome of surgery. Cote et al^4^ based their guidelines for rehabilitation following ACJR on the tissuehealing time frames of tendon healing in a bone tunnel. Construct failure through pull-out of the tendon from the tunnel occured less than 12 weeks post-op while failure at the midsubstance of the tendon occured at 12 weeks or more. Therefore, isotonic strength activities are not started for 12 weeks because of concern about the ability of the surgical construct to tolerate a repetitively applied load. In our case, it was clear that strengthening exercises were started too early and this caused the construct to fail. A similar failure was also reported in another study^5^ where the patient did not comply with rehabilitation protocol.

In summary, ACJR using semitendinosus tendon graft has been proven to be superior biochemically, clinically, and radiologically. However, the patient should be compliant to the rehabilitation protocol prescribed and this involves communicating and working together with the sports physicians and physiotherapists to ensure an optimal outcome of the reconstructive procedure. Failure of this, as shown in this case, resulted in failure of the surgery.

**Fig. 1a:**
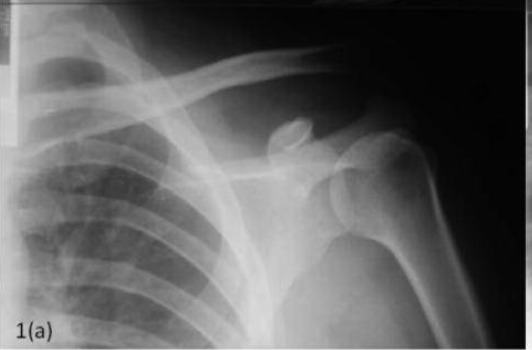
: Pre operative radiograph showing superior displacement of he clavicle and undisplaced distal clavicle fracture.

**Fig. 1b:**
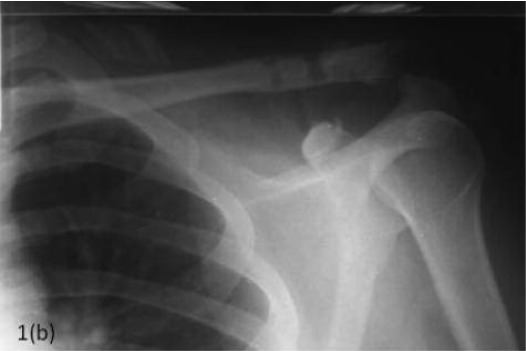
: Immediate post operative radiograph of the shoulder following operative reduction and ligament reconstruction.

**Fig. 2a:**
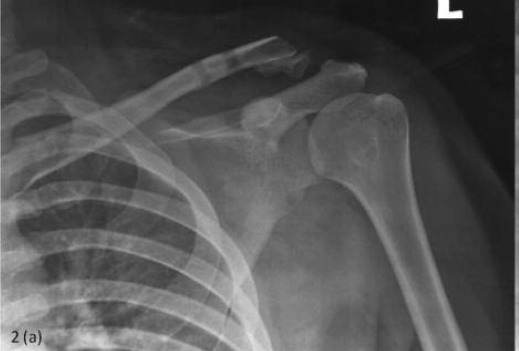
: 10 weeks post operative radiograph showing displaced distal clavicle fracture.

**Fig. 2b:**
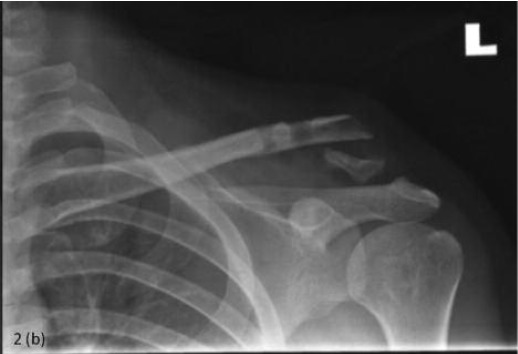
: 5 months post operative radiograph showing nonunion of distal clavicle fracture.

**Fig. 3:**
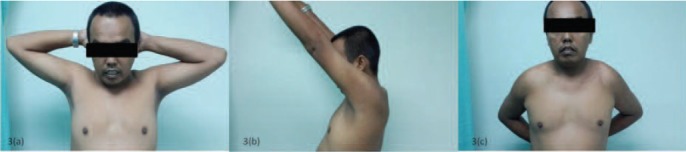
: Five months post operative pictures showing range of motion of both shoulders.
